# Impairment of health-related quality of life among people with type 2 diabetes and advanced liver fibrosis

**DOI:** 10.1038/s41598-024-72105-8

**Published:** 2024-09-17

**Authors:** Maurice Michel, Jesús Funuyet-Salas, Michelle Doll, Saleh A. Alqahtani, Angelo Armandi, Christian Labenz, Peter R. Galle, Jörn M. Schattenberg

**Affiliations:** 1 Department of Internal Medicine II, University Medical Centre Saarland, Homburg, Germany; 2 Department of Internal Medicine I, University Medical Centre Mainz, Mainz, Germany; 3 Loyola University Andalusia, Sevilla, Spain; 4 Liver Transplantation Center, King Faisal Specialist Hospital & Research Center, Riyadh, Saudi Arabia; 5 Division of Gastroenterology and Hepatology, Johns Hopkins University, Baltimore, MD USA; 6 Department of Medical Sciences, University of Turin, Turin, Italy; 7 Saarland University, Saarbrücken, Germany

**Keywords:** MASLD, Advanced fibrosis, T2DM, Obesity, HRQL, Hepatology, Type 2 diabetes, Obesity

## Abstract

People with type 2 diabetes mellitus (T2DM) show a high prevalence of steatotic liver disease (SLD), and especially metabolic dysfunction-associated steatotic liver disease (MASLD), with liver fibrosis. Their health-related quality of life (HRQL) is affected by multiple in part overlapping factors and aggravated by metabolic and liver-related comorbidities, including liver fibrosis stage. The aim of this study was to investigate the effect size of advanced fibrosis (AF) on the HRQL in people with T2DM. A total of 149 individuals with T2DM treated at a primary care provider within the German disease management program (DMP) were included in the final analysis. Vibration-controlled transient elastography (VCTE) was used to non-invasively detect steatosis and AF. The EQ-5D-3L questionnaire was used to assess the HRQL. Uni- and multivariable linear regression models were used to identify independent predictors of impaired HRQL. The majority was male (63.1%), and the median age was 67 years (IQR 59; 71). In the entire cohort, the prevalence of MASLD and AF was 70.7% and 19.5%, respectively. People with T2DM and AF had an overall lower HRQL in comparison to those without AF (p < 0.001). Obesity (β: − 0.247; 95% CI − 0.419, − 0.077) and AF (β: − 0.222; 95% CI − 0.383, − 0.051) remained independent predictors of a poor HRQL. In turn, T2DM-related comorbidities were not predictive of an impaired HRQL. Obesity and AF negatively affect the HRQL in patients with SLD and T2DM in primary care. Awareness of liver health and specific interventions may improve patient-reported and liver-related outcomes in people with T2DM.

## Introduction

Steatotic liver disease (SLD) and especially metabolic dysfunction-associated steatotic liver disease (MASLD) has become the most common chronic liver disease with an estimated global prevalence of 30%^1^. Major risk factors for developing MASLD are type 2 diabetes mellitus (T2DM) and obesity, with a markedly elevated prevalence of MASLD if these conditions are present^2–4^. Along the disease course, MASLD can progress to metabolic dysfunction-associated steatohepatitis (MASH), with increasing scarring of liver tissue resulting in liver fibrosis^5^. Progression of liver fibrosis can lead to advanced fibrosis (AF; F3) and even liver cirrhosis (F4), with increased morbidity and mortality^6^. As a result of the increasing burden of chronic liver disease related to MASLD, non-invasive tests (NITs), including vibration-controlled transient elastography (VCTE, Fibroscan^®^), have been developed to identify patients at risk in need of more intensive care and surveillance^7^. However, only few data on the use of VCTE is available from primary care settings, although the majority of patients with T2DM are routinely seen by primary care providers^8^.

An important aspect in the care of patients is the health-related quality of life (HRQL)—a term that describes the patient’s physical, mental, and social functioning, individual perception of their health status, and the overall well-being associated with the disease. Several questionnaires have been developed for the assessment of the HRQL. A generic questionnaire is the EQ-5D-3L that can assess the current health state, and provide an estimate of the overall HRQL^9^. The EQ-5D-3L consists of five dimensions that capture various aspects of someone’s health (mobility, self-care, usual activities, pain/discomfort, and anxiety/depression), with three levels of response (no problems, moderate problems, or extreme problems) each. It is a validated tool for the assessment of the HRQL that has also been used in the context of T2DM or SLD/MASLD^10,11^. Analyzing the HRQL has major implications for treatment adherence and success, especially within the context of chronic conditions requiring long-term care such as T2DM and MASLD^12^. Patients with MASLD and higher disease stages, including MASH and fibrosis, report a lower HRQL^10,13^. In patients with T2DM, impairment of HRQL is often associated with T2DM-related comorbidities, including microvascular and macrovascular complications^14–16^. However, despite the high prevalence of SLD and AF in people with T2DM, the independent effect size of the liver phenotype on the HRQL remains to be established. Therefore, the aim of this prospective study was to investigate the effect size of AF on the HRQL in people with T2DM.

## Methods

### Study design and population

A total of 153 participants with a diagnosis of T2DM were enrolled at a primary care provider (Diabetology Practice Mainz) with a focus on T2DM in Mainz, Germany, between 2019 and 2021 (Supplementary Fig. 1). These individuals were recruited during their routine visits within the T2DM disease management program (DMP). The DMP is a specifically designed program for the routine assessment of T2DM-related comorbidities, including microvascular and macrovascular complications, to decrease their associated mortality^17^. All participants had to be at least 18 years of age and provide written informed consent before study inclusion. Only participants with a diagnosis of T2DM were eligible for study inclusion with no specific differences in disease severity, treatment, or the number of related comorbidities. Due to missing data or invalid VCTE measurements, four individuals were excluded from the final analysis (n = 149). Anthropometric data, patient history, and laboratory values were obtained from each participant at study inclusion. Body mass index (BMI) was calculated using height and weight (BMI = weight [kg]/height^2^ [m^2^]). Waist circumference (cm) was assessed at study inclusion. Metabolic syndrome (MetS) was defined according to the international diabetes federation^18^. The alcohol use disorders identification test (AUDIT) was used to screen for alcohol consumption, and a score ≥ 8 was considered at least harmful alcohol consumption^19^.

### Non-invasive assessment of hepatic steatosis (HS) and advanced fibrosis (AF)

Hepatic steatosis (HS) for a diagnosis of SLD and advanced fibrosis (AF) were non-invasively assessed using vibration-controlled transient elastography (VCTE; Fibroscan^®^ 430 mini) and defined as a controlled attenuation parameter (CAP) of  ≥ 275 and a liver stiffness measurement (LSM) of ≥ 12, respectively. CAP was assessed by a standard exam of 10 measurements. The cutoffs were chosen based on recent EASL guidelines on the use of NITs and current guidelines on the care of MASLD^20,21^. Either the M or XL probe was used as recommended by the device for each participant. MASLD was defined according to current practice guidelines after the exclusion of high alcohol consumption (based on AUDIT) or other secondary causes of SLD^21^.

### Assessment of health-related quality of life (HRQL)

The validated German version of the EQ-5D-3L questionnaire was used for the assessment of the HRQL. The EQ-5D is a generic measure to quantify an individual’s HRQL, which has also been used in people with T2DM and patients with chronic liver disease, including MASH with advanced fibrosis^10,11,22^. The questionnaire contains five dimensions, mobility, self-care, usual activities, pain/discomfort, and anxiety/depression, that each captures various aspects of the HRQL. Each dimension has three response levels ranging from no problems, moderate problems to extreme problems. The EQ VAS indicates the overall current health, ranging from 0 (the worst health) to 100 (the best health). In turn, the time trade-off (TTO) index value represents an overall score of the five dimensions based on a country-specific value set that reflects the respective health state of that country. For this study, the German value set was used^23^. Higher scores on the five dimensions indicate a lower HRQL, whereas higher scores on the VAS and TTO indicate a better HRQL.

### Ethics

All patients provided written informed consent. The study was conducted according to the ethical guidelines of the 1975 Declaration of Helsinki (sixth revision, 2008). The study protocol was approved by the ethics committee of the Landesärztekammer Rhineland–Palatinate [Nr. 873.199.10 (7208)].

### Statistics

Descriptive variables are presented as median values with interquartile ranges (IQR 25th; 75th) or mean values with SD. Categorical variables are shown as frequencies with percentages. For the comparison of differences between groups of categorical variables, the Mann–Whitney *U* test was used. The chi-squared test was applied for the comparison of two or more patient groups. All tests were two-tailed, and statistically significant values were defined as p < 0.05. Linear regression models were built to identify independent predictors of an impaired HRQL (TTO, VAS). All variables with a p-value of < 0.05 in the univariable analysis, and adjusted for age and sex, were then analyzed in a multivariable linear regression model. Cohen’s *d* (for continuous variables) and *w* (for categorical variables) were computed as effect size indexes. Effect sizes are defined as null (*d* < 0.2; *w* < 0.1), small (*d* ≥ 0.2; *w* ≥ 0.1), medium (*d* ≥ 0.5; w ≥ 0.3), or large (d ≥ 0.8; w ≥ 0.5)^24^. For all data analyses and statistical tests, IBM SPSS Statistic Version 23.0 (IBM Corp.) was used. Microsoft Excel 2016 (Microsoft Corp.) or Microsoft PowerPoint 2016 (Microsoft Corp.) were used for all figures.

## Results

### Demographic and clinical characteristics

The median age in this cohort was 67 (IQR 59; 71) years and 36.9% were females. Advanced fibrosis (AF) was evident in 19.5% (n = 29). The prevalence of SLD was 77.9% (n = 116), of whom the majority had MASLD (n = 104, 70.7%). Cardiovascular disease (CVD) was more prevalent in people with AF (p = 0.002, d = 0.256). The median CAP was 336 dB/m (IQR 280.5; 371.0) in the entire cohort, with a significantly higher median CAP in those with AF compared to no AF (p = 0.043, d = −0.481). Metabolic risk factors were more common in the subgroup with AF. Higher median GGT lab values were detected in those with AF (p < 0.001, d = −0.518). More individuals were on insulin as part of their T2DM-related treatment in those with AF compared to no AF (p = 0.008, d = 0.217). No major differences were seen between males and females, although a higher alcohol consumption was more evident in males (p = 0.031, d = −0.177; Supplementary Table 1). Baseline characteristics and a comparison between people with T2DM presenting with and without AF are summarized in Table 1.

**Table 1:** Demographics, clinical characteristics, and comparison between AF and no AF.

Variable	Total cohort (n = 149)	No AF (n = 120, 80.5%)	AF (n = 29, 19.5%)	p-value	Effect sizes
	n (% or IQR)	n (% or IQR)	n (% or IQR)		Cohen’s d or w
General characteristics					
Age in years	67.0 (59.0; 71.0)	67.0 (59.0; 71.0)	64.0 (58.5; 69.0)	0.150	0.345 S
Time since diagnosis	11.0 (6.0; 18.5)	11.0 (5.3; 17.0)	12.0 (7.0; 21.5)	0.330	− 0.176 N
Sex, female	55 (36.9)	42 (35)	13 (44.8)	0.325	0.081 N
History of cancer	13 (8.7)	10 (8.3)	3 (10.3)	0.730	0.028 N
Harmful alcohol consumption (AUDIT ≥ 8) n = 148	12 (8.1)	8 (6.7)	4 (14.3)	0.184	0.109 S
VCTE					
CAP (dB/m)	336 (280.5; 371.0)	327.5 (274.0; 368.8)	361.0 (301.5; 387.5)	**0.043**	− 0.481 S
SLD (CAP ≥ 275 dB/m)	116 (77.9)	89 (74.2)	27 (93.1)	**0.028**	0.181 S
MASLD n = 147	104 (70.7)	81 (67.5)	23 (85.2)	0.068	0.151 S
LSM (kPa)	7.2 (5.6; 10.9)	6.4 (5.3; 8.4)	18 (14.2; 20.5)	** < 0.001**	− 1.358 L
Metabolic comorbidities					
BMI (kg/m^2^)	31.8 (27.6; 35.5)	30.3 (27.1; 34.3)	35.3 (33.5; 42.2)	** < 0.001**	− 1.057 L
Obesity (> 30 kg/m^2^)	89 (59.7)	62 (51.7)	27 (93.1)	**0.001**	0.335 M
Waist circumference (cm)	110 (100.0; 123.5)	106.5 (99.0; 120.8)	124.0 (114.5; 138.0)	** < 0.001**	− 1.189 L
Hyperlipidemia	93 (62.4)	75 (62.5)	18 (62.1)	0.966	0.056 N
Arterial hypertension	134 (89.9)	106 (88.3)	28 (96.6)	0.187	0.108 S
MetS	89 (59.7)	62 (51.7)	27 (93.1)	** < 0.001**	0.335 M
Laboratory values					
GGT (U/l) n = 148	37.5 (26.3; 56.8)	34.0 (26.0; 48.0)	60.0 (37.0; 83.5)	** < 0.001**	− 0.518 M
TG Triglycerides (mg/dl) n = 147	165 (112; 249)	162.5 (105.0; 245.3)	184.0 (119.5; 299.5)	0.158	− 0.182 N
TC (mg/dl) n = 148	190.0 (156.0; 219.8)	190.0 (155.0; 222.0)	189.0 (157.5; 213.5)	0.519	0.198 N
Platelets (/nl) n = 148	221.0 (193.0; 226.3)	221.0 (193.0; 273.0)	218.0 (186.0; 247.0)	0.614	0.043 N
HbA1c (%) n = 148	7.0 (6.5; 7.7)	6.9 (6.4; 7.6)	7.5 (6.7; 8.4)	**0.031**	− 0.418 S
T2DM-related comorbidities					
CVD	55 (36.9)	37 (30.8)	18 (62.1)	**0.002**	0.256 S
CKD	49 (32.9)	41 (34.2)	8 (27.6)	0.498	− 0.055 N
Polyneuropathy	47 (31.5)	35 (29.2)	12 (41.4)	0.204	0.104 S
Retinopathy	14 (9.4)	7 (5.8)	7 (24.1)	**0.002**	0.248 S
DFS	19 (12.8)	13 (10.8)	6 (20.7)	0.153	0.117 S
T2DM-related medication					
Metformin	92 (61.7)	74 (61.7)	18 (62.1)	0.968	0.003 N
Insulin	75 (50.3)	54 (45.0)	21 (72.4)	**0.008**	0.217 S
SGLT2 inhibitor	22 (14.8)	15 (12.5)	7 (24.1)	0.113	0.130 S
No treatment	16 (10.7)	15 (12.5)	1 (3.4)	0.158	− 0.116 S

*AF* advanced fibrosis, *AUDIT* alcohol use disorders identification test, *VCTE* vibration-controlled transient elastography, *CAP* controlled attenuation parameter, *MASLD* metabolic dysfunction-associated steatotic liver disease, *LSM* liver stiffness measurement, *BMI* body mass index, *MetS* metabolic syndrome, *GGT* gamma-glutamyl transferase, *SLD* steatotic liver disease, *TG* triglycerides, *TC* total cholesterol, *CVD* cardiovascular disease, *CKD* chronic kidney disease, *DFS* diabetic foot syndrome.

Effect sizes: N, null; S, small; M, medium; L, large. Data are expressed as numbers, median, percentage (%), or interquartile ranges (IQR 25th; 75th).

P-values refer to the comparison between no AF vs. AF. Boldface indicates statistical significance. A p-value < 0.05 was considered statistically significant.

### HRQL in people with T2DM

**Fig. 1:**
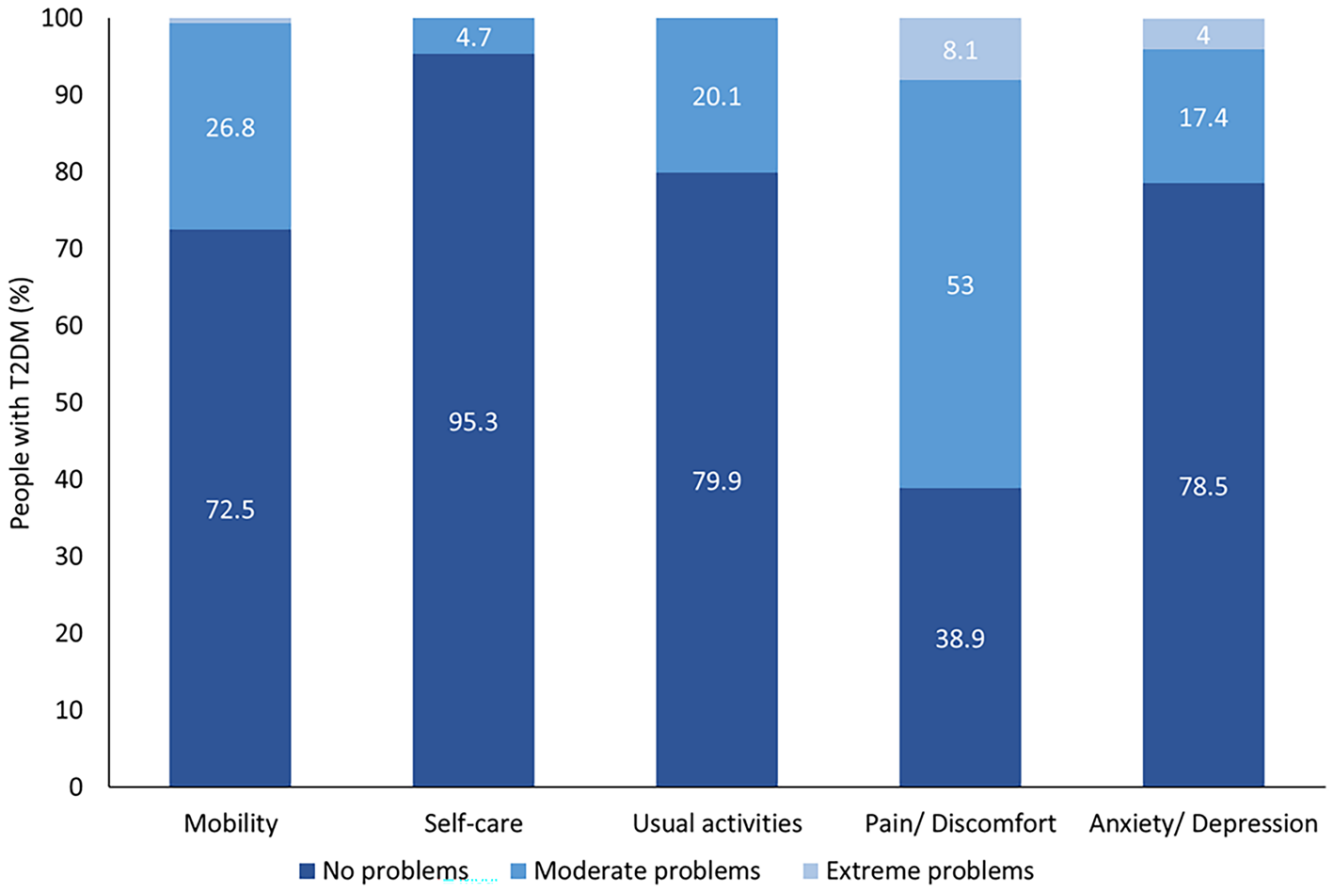
Distribution of the EQ-5D-3L dimensions in people with T2DM presenting with advanced fibrosis (AF) or without advanced fibrosis (no AF). The EQ-5D-3L consists of five dimensions: mobility, self-care, usual activities, pain/discomfort, and anxiety/depression. Each of these dimensions is divided into three levels of perceived problems: no problems, moderate problems, or extreme problems.

The mean EQ-5D-3L VAS and TTO index value was 71.9 ± 18.4 and 0.85 ± 0.21, respectively. The highest mean scores were seen in the pain/discomfort (1.70 ± 0.61) and mobility (1.28 ± 0.47) dimensions, whereas the lowest mean score was detected in the self-care (1.05 ± 0.21) dimension (Table 2). A high proportion of people with T2DM reported moderate and extreme problems in the pain/discomfort dimension in comparison to the other dimensions (Fig. 1). The anxiety/depression dimension was the only dimension to show a significant difference between males and females (1.20 ± 0.49 vs. 1.34 ± 0.55, p = 0.043, d = −0.269) (Supplementary Table 2).

**Table 2:** Mean scores of the EQ-5D-3L questionnaire.

Variable	Total cohort	No AF	AF	p-value	Effect sizes Cohen’s d
EQ-5D-3L					
Mobility n = 148	1.28 ± 0.47	1.22 ± 0.41	1.55 ± 0.57	**0.001**	− 0.665 M
Self-care	1.05 ± 0.21	1.03 ± 0.18	1.10 ± 0.31	0.110	− 0.276 S
Usual activities	1.20 ± 0.40	1.17 ± 0.37	1.34 ± 0.48	**0.032**	− 0.397 S
Pain/discomfort n = 147	1.70 ± 0.61	1.64 ± 0.59	1.93 ± 0.65	**0.027**	− 0.467 S
Anxiety/depression	1.26 ± 0.52	1.21 ± 0.45	1.45 ± 0.74	0.102	− 0.392 S
VAS n = 143	71.9 ± 18.4	74.8 ± 16.4	59.9 ± 21.3	** < 0.001**	0.784 M
TTO index-value n = 146	0.85 ± 0.21	0.88 ± 0.17	0.71 ± 0.29	**0.001**	0.715 M

Data are expressed as means with standard deviation (± SD). Data are expressed as means with standard deviation. Boldface indicates statistical significance. Effect sizes: S, small; M, medium.

A p-value < 0.05 was considered statistically significant.

### Comparison of the HRQL between AF and without AF

**Fig. 2:**
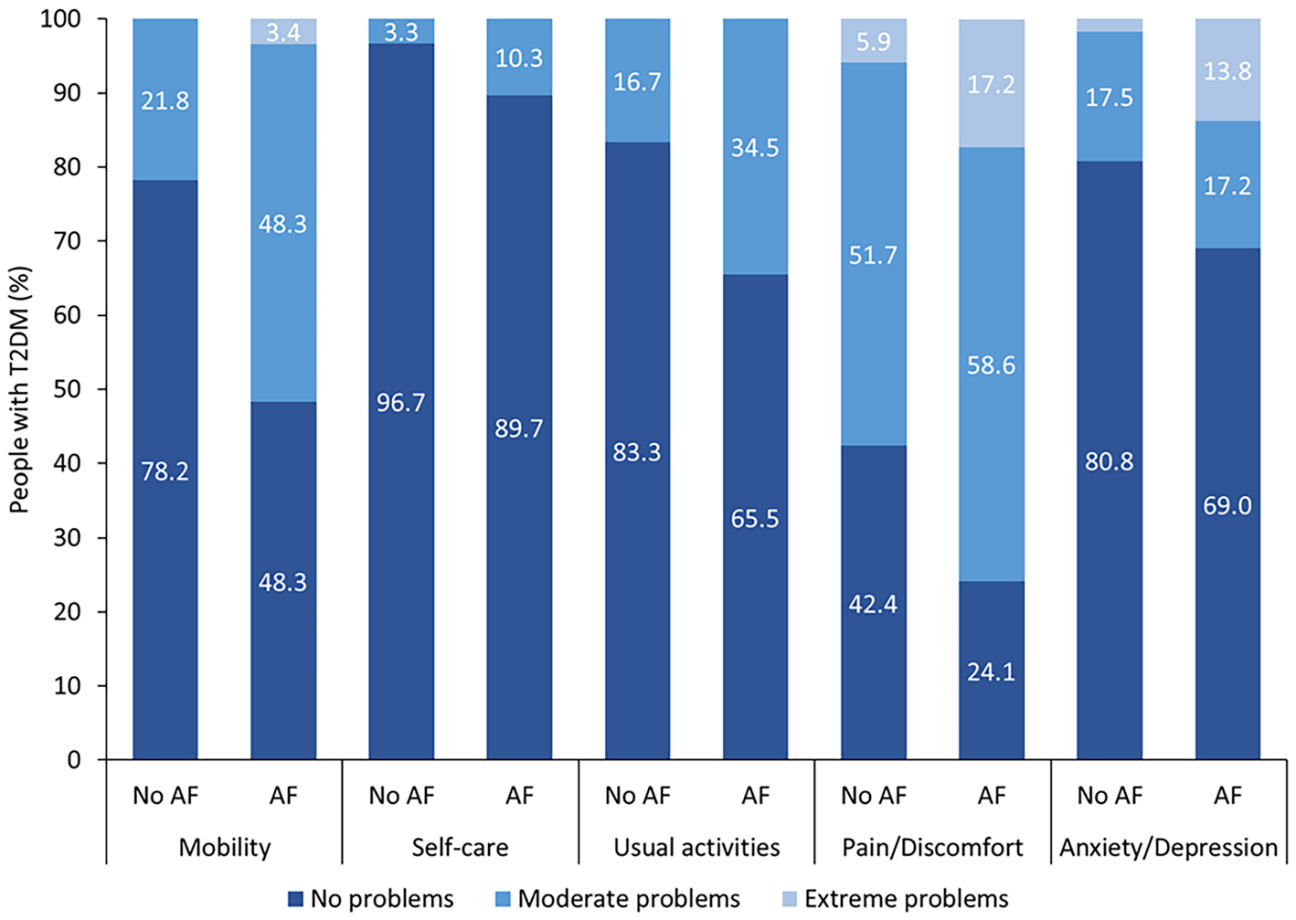
Distribution of the EQ-5D-3L dimensions in people with T2DM in the entire cohort. The EQ-5D-3L consists of five dimensions: mobility, self-care, usual activities, pain/discomfort, and anxiety/depression. Each of these dimensions is divided into three levels of perceived problems: no problems, moderate problems, or extreme problems.

The HRQL was overall lower in AF (VAS: no AF 74.8 ± 16.4 vs. AF 59.9 ± 21.3, p < 0.001, d = 0.784; TTO: no AF 0.88 ± 0.17 vs. AF 0.71 ± 0.29, p = 0.001, d = 0.715). Significantly higher mean scores for mobility (1.55 ± 0.57, p = 0.001, d = −0.665), usual activities (1.34 ± 0.48, p = 0.032, d = −0.397), and pain/discomfort (1.93 ± 0.65, p = 0.027, d = −0.467) dimensions were seen in those with AF (Table 2). Although the mean score of the anxiety/depression dimension was higher in AF, no significant difference was seen in the comparison to those without AF. In the pain/discomfort and mobility dimensions, more individuals with AF reported moderate and extreme problems compared to no AF. Furthermore, those with AF showed the second-highest distribution of extreme problems in the anxiety/depression dimension (Fig. 2). In addition, a comparison between people with T2DM presenting with SLD and without SLD and/or obesity is shown in Supplementary Table 3 and Supplementary Table 4, respectively.

### Clinical predictors of impaired HRQL in people with T2DM

Two linear regression models, based on the TTO index value and the VAS, were built to identify independent predictors of impaired HRQL in people with T2DM (Table 3**, **Table 4). The variables time since diagnosis, metabolic risk factors (obesity, waist circumference, MetS), liver-related comorbidities (SLD, AF), T2DM-related comorbidities (CVD, retinopathy, polyneuropathy, and DFS), and treatment with insulin were all associated with a lower HRQL in the univariable analysis. In the multivariable linear regression analysis, obesity (β: −0.225; 95% CI −0.399, −0.050) and AF (β: −0.171; 95% CI −0.322, −0.001) remained the only independent predictors of a poor HRQL (Table 3).

**Table 3:** Clinical predictors of impaired HRQL in T2DM according to TTO index value.

Variables	Univariable analysis			Multivariable analysis^†^		
	β	95% CI	p-value	β	95% CI	p-value
General characteristics						
Age	− 0.150	− 0.339, 0.013	0.070	− 0.144	− 0.346, 0.035	0.108
Time since diagnosis	** − 0.236**	** − 0.386, − 0.074**	**0.004**	− 0.069	− 0.250, 0.117	0.472
Sex, female	− 0.072	− 0.237, 0.093	0.390	− 0.129	− 0.285, 0.026	0.102
History of cancer	0.064	− 0.093, 0.211	0.443			
Harmful alcohol consumption	0.013	− 0.151, 0.177	0.874			
Metabolic comorbidities						
Obesity	** − 0.298**	** − 0.455, − 0.141**	** < 0.001**	** − 0.225**	** − 0.399, − 0.050**	**0.012**
Waist circumference	** − 0.329**	** − 0.481, − 0.172**	** < 0.001**			
Arterial hypertension	− 0.033	− 0.211, 0.142	0.696			
Hyperlipidemia	0.104	− 0.059, 0.267	0.211			
Metabolic syndrome	** − 0.298**	** − 0.455, − 0.140**	** < 0.001**			
VCTE						
SLD	** − 0.175**	** − 0.343, − 0.013**	**0.035**	− 0.036	− 0.204, 0.131	0.667
AF	** − 0.318**	** − 0.460, − 0.157**	** < 0.001**	** − 0.171**	** − 0.332, − 0.001**	**0.049**
Laboratory values						
HbA1c	− 0.061	− 0.256, 0.118	0.466			
GGT	− 0.024	− 0.223, 0.166	0.773			
T2DM-related comorbidities						
CVD	** − 0.248**	** − 0.393, − 0.085**	**0.003**	− 0.104	− 0.265, 0.064	0.228
CKD	− 0.073	− 0.236, 0.090	0.379			
Retinopathy	** − 0.194**	** − 0.339, − 0.031**	**0.019**	− 0.068	− 0.221, 0.091	0.411
Polyneuropathy	** − 0.250**	** − 0.406, − 0.090**	**0.002**	− 0.094	− 0.275, 0.089	0.315
DFS	** − 0.197**	** − 0.322, − 0.033**	**0.017**	− 0.046	− 0.199, 0.114	0.592
T2DM-related medication						
Metformin	0.055	− 0.108, 0.218	0.507			
Insulin	** − 0.228**	** − 0.387, − 0.068**	**0.006**	− 0.028	− 0.206, 0.151	0.761
SGLT2 Inhibitor	− 0.053	− 0.206, 0.106	0.525			
No treatment	0.105	− 0.054, 0.249	0.205			

Univariable and multivariable linear regression analysis is shown. With all factors showing a p-value < 0.05 and adjusted for age and sex, a multivariable linear regression model was built. The variables waist circumference and MetS were excluded from the multivariable analysis to avoid multicollinearity. Confidence interval (CI) and beta (β) show each standardized values. Boldface indicates statistical significance.

A p-value < 0.05 was considered statistically significant.

^**†**^Multivariable linear regression analysis (n = 146): age, sex, time since diagnosis, obesity, SLD, AF, CVD, retinopathy, polyneuropathy, DFS, insulin.

**Table 4:** Clinical predictors of impaired HRQL in T2DM according to VAS.

Variables	VAS					
	Univariable analysis			Multivariable analysis^†^		
	β	95% CI	p-value	β	95% CI	p-value
General characteristics						
Age	− 0.140	− 0.331, 0.027	0.095	** − 0.171**	** − 0.369, − 0.003**	**0.046**
Time since diagnosis	− 0.106	− 0.265, 0.058	0.208			
Sex, female	0.093	− 0.073, 0.262	0.267	0.052	− 0.106, 0.210	0.514
History of cancer	0.033	− 0.122, 0.182	0.696			
Harmful alcohol consumption	− 0.061	− 0.224, 0.104	0.469			
Metabolic comorbidities						
Obesity	** − 0.292**	** − 0.454, − 0.134**	** < 0.001**	** − 0.247**	** − 0.419, − 0.077**	**0.005**
Waist circumference	** − 0.361**	** − 0.528, − 0.210**	** < 0.001**			
Arterial hypertension	− 0.155	− 0.339, 0.010	0.065			
Hyperlipidemia	0.097	− 0.069, 0.262	0.251			
MetS	** − 0.292**	** − 0.453, − 0.133**	** < 0.001**			
VCTE						
SLD	− 0.078	− 0.252, 0.091	0.354			
AF	** − 0.322**	** − 0.468, − 0.160**	** < 0.001**	** − 0.222**	** − 0.383, − 0.051**	**0.011**
Laboratory values						
HbA1c	− 0.079	− 0.286, 0.102	0.351			
GGT	− 0.057	− 0.262, 0.129	0.501			
T2DM-related comorbidities						
CVD	** − 0.247**	** − 0.393, − 0.082**	**0.003**	− 0.139	− 0.298, 0.031	0.110
CKD	− 0.056	− 0.217, 0.108	0.509			
Retinopathy	− 0.116	− 0.265, 0.047	0.168			
Polyneuropathy	** − 0.202**	** − 0.358, − 0.038**	**0.015**	− 0.031	− 0.212, 0.151	0.740
DFS	** − 0.175**	** − 0.311, − 0.010**	**0.037**	− 0.043	− 0.195, 0.116	0.616
T2DM-related medication						
Metformin	− 0.063	− 0.228, 0.102	0.452			
Insulin	** − 0.175**	** − 0.337, − 0.011**	**0.037**	− 0.005	− 0.170, 0.161	0.954
SGLT2 Inhibitor	− 0.067	− 0.219, 0.093	0.426			
No treatment	0.107	− 0.055, 0.257	0.202			

Univariable and multivariable linear regression analysis is shown. With all factors showing a p-value < 0.05 and adjusted for age and sex, a multivariable linear regression model was built. The variables waist circumference and MetS were excluded from the multivariable analysis to avoid multicollinearity. Confidence interval (CI) and beta (β) show each standardized values. Boldface indicates statistical significance.

A p-value < 0.05 was considered statistically significant.

^**†**^Multivariable linear regression analysis (n = 146): age, sex, obesity, AF, CVD, polyneuropathy, DFS, insulin.

Similar results were seen with the VAS. Here, the variables obesity, waist circumference, MetS, AF, CVD, polyneuropathy, DFS, and insulin were associated with a lower HRQL. Age (β: −0.171; 95% CI −0.369, −0.003), obesity (β: −0.247; 95% CI −0.419, -0.077), and AF (β: −0.222; 95% CI −0.383, −0.051) remained the only independent predictors a poor HRQL in the multivariable linear regression analysis (Table 4).

## Discussion

In this study, we aimed to analyze the effect size of AF on the HRQL in people with T2DM in primary care in the German DMP using the EQ-5D-3L questionnaire. Overall, the burden of liver-related comorbidities, including SLD and AF, is high in these individuals with a significant impact on the HRQL. The mobility and pain/discomfort dimensions imposed the highest burden. Especially those with AF showed the lowest scores on the VAS and the TTO index value. Although the presence of T2DM-related comorbidities was in part negatively affecting the HRQL, AF, and obesity remained the only independent predictors of an impaired HRQL in this cohort. Thus, obesity and more advanced liver disease may impose a higher impairment of HRQL than other well-known T2DM-related side effects. This has important implications for the consideration of SLD/MASLD and AF in the routine assessment of people with T2DM in disease management programs.

Obesity and AF remained the only independent predictors of HRQL impairment as measured by the VAS and the TTO index value in this cohort. Other studies have also reported the negative impact of higher fibrosis stages, including AF, on the HRQL in patients with MASLD^10,25^. Moreover, especially people with T2DM and obesity had a greater impairment of the HRQL compared to those without obesity^26^. In comparison to AF, the detection of SLD was not independently associated with a lower HRQL. Chronic liver diseases, such as MASLD, may lack specific symptoms, especially in earlier stages without MASH and fibrosis. Moreover, obesity may reflect a more obvious sign of a metabolically ill phenotype than MASLD itself, with the result of body dissatisfaction, stigmatization, and discrimination^27^. Furthermore, obesity can also impose restrictions on routine activities due to physical inability^28^. In this context, the most burdensome dimensions were mobility, usual activities, and pain/discomfort in people with T2DM and AF/obesity. These findings are in line with previous analyses in chronic conditions other than T2DM^29^. Physical activity is an important determinant of the HRQL in T2DM and MASLD^30,31^. This is highly important in the context of lacking pharmacotherapy in MASLD, with lifestyle interventions aiming at weight reduction as the cornerstone of treatment. Obesity and AF could be both self-reinforcing factors from various perspectives with negative effects on the HRQL.

People with T2DM report an overall lower HRQL in comparison to the general population^32^. The high prevalence of obesity in these individuals may add to the burden of a decreased HRQL, as our study and other studies confirm^33^. More interestingly, the T2DM-related comorbidities investigated in this study did not remain predictive of a lower HRQL. Previous analyses highlighted the negative effects of comorbidities on the HRQL in people with T2DM^14–16^. However, none of these studies have specifically addressed liver-related comorbidities despite the high prevalence in these patients^2^. In this cohort, a higher prevalence of T2DM-related comorbidities was seen in the subgroup with AF. The presence of AF resembles an overall morbid phenotype with a high risk of several other hepatic- and extrahepatic complications, and high mortality^6^. In a large study of people with T2DM in primary care, obesity did not remain an independent predictor of a lower HRQL which is in contrast to our findings. It was rather the number of symptomatic comorbidities associated with T2DM that showed a high impact on HRQL^16^. While we did not analyze the number of comorbidities specifically, we argue that the strong effect of AF on the HRQL in our study could be an expression of these multiple in part overlapping factors, especially within the context of T2DM. Implementing a routine assessment of liver fibrosis in people with T2DM may aid in identifying those with an overall higher disease burden and a lower HRQL.

Other studies have shown a higher prevalence and risk of anxiety and depression in people with T2DM and those with MASLD^34,35^. In this study, anxiety or depression was not specifically assessed. The anxiety/depression dimension of the EQ-5D-3L showed no impairment in the entire cohort and differences between the subgroups, although 31% expressed moderate to extreme problems in this dimension if AF was present. As reported by the VAS, age remained predictive of poor HRQL along with obesity and AF. Other studies have shown that VAS decreases with higher age, which may be related to the addition of more age-related conditions along with common T2DM-related comorbidities^36^. Furthermore, no difference in the HRQL between males and females was detected, contradictory to other studies highlighting a lower HRQL in females with advanced stages of MASLD^13^.

### Strengths & limitations

A limitation of this study is the highly selected study population and in this regard the lack of a control group without T2DM. However, we here present data from a primary care setting of people with T2DM and with the inclusion of major related comorbidities. In this context, the analyses were adjusted for relevant confounders of an impaired HRQL along with obesity and AF, leading to greater generalizability of these findings in populations with T2DM. Yet, AF is often associated with metabolic risk factors and other comorbidities, and thus multicollinearity in the multivariable analyses cannot entirely be ruled out which may limit the results of this study. Besides MASLD, we have also included cases of SLD with harmful alcohol consumption (alcohol-related liver disease). However, the majority of this cohort presented with MASLD, and the inclusion of harmful alcohol consumption had the purpose of ruling out this confounder on HRQL. The monocentric nature of this study without longitudinal assessment limits the ability to assess causation. Moreover, SLD and AF were defined non-invasively according to VCTE thresholds with the possibility of false negative or false positive findings. Despite inaccuracies of VCTE and CAP for the detection of hepatic steatosis, the threshold used in this study of ≥ 275 dB/m has a sensitivity and positive predictive value of over 90%^20^.

## Conclusions

This study identified obesity and AF as independent predictors of an impaired HRQL in people with T2DM in primary care. These findings highlight the importance of chronic liver disease with AF in the assessment of this high-risk population. Awareness of liver health and specific interventions may improve patient-reported and liver-related outcomes in people with T2DM.

## Supplementary Information

Supplementary Figure 1.

Supplementary Tables.

## Data Availability

The data presented in this study are available on request from the corresponding author.

## Abbreviations

SLD: Steatotic liver disease
MASLD: Metabolic dysfunction-associated steatotic liver disease
MASH: Metabolic dysfunction-associated steatohepatitis
AF: Advanced fibrosis
T2DM: Type 2 diabetes mellitus
MetS: Metabolic syndrome
NITs: Non-invasive tests
VCTE: Vibration-controlled transient elastography
HRQL: Health-related quality of life
DMP: Disease management program
BMI: Body mass index
AUDIT: Alcohol use disorders identification test
HS: Hepatic steatosis
CAP: Controlled attenuation parameter
LSM: Liver stiffness measurement
TTO: Time trade-off
VAS: Visual analog scale
CVD: Cardiovascular disease
CKD: Chronic kidney disease
DFS: Diabetic foot syndrome
GGT: Gamma-glutamyl transferase
TG: Triglycerides
TC: Total cholesterol
