# Supplementary figures and images for: Impairment of health-related quality of life among people with type 2 diabetes and advanced liver fibrosis
Source: Sci Rep. 2024 Sep 17;14:21650. doi: 10.1038/s41598-024-72105-8 (PMC11408596; doi:10.1038/s41598-024-72105-8)

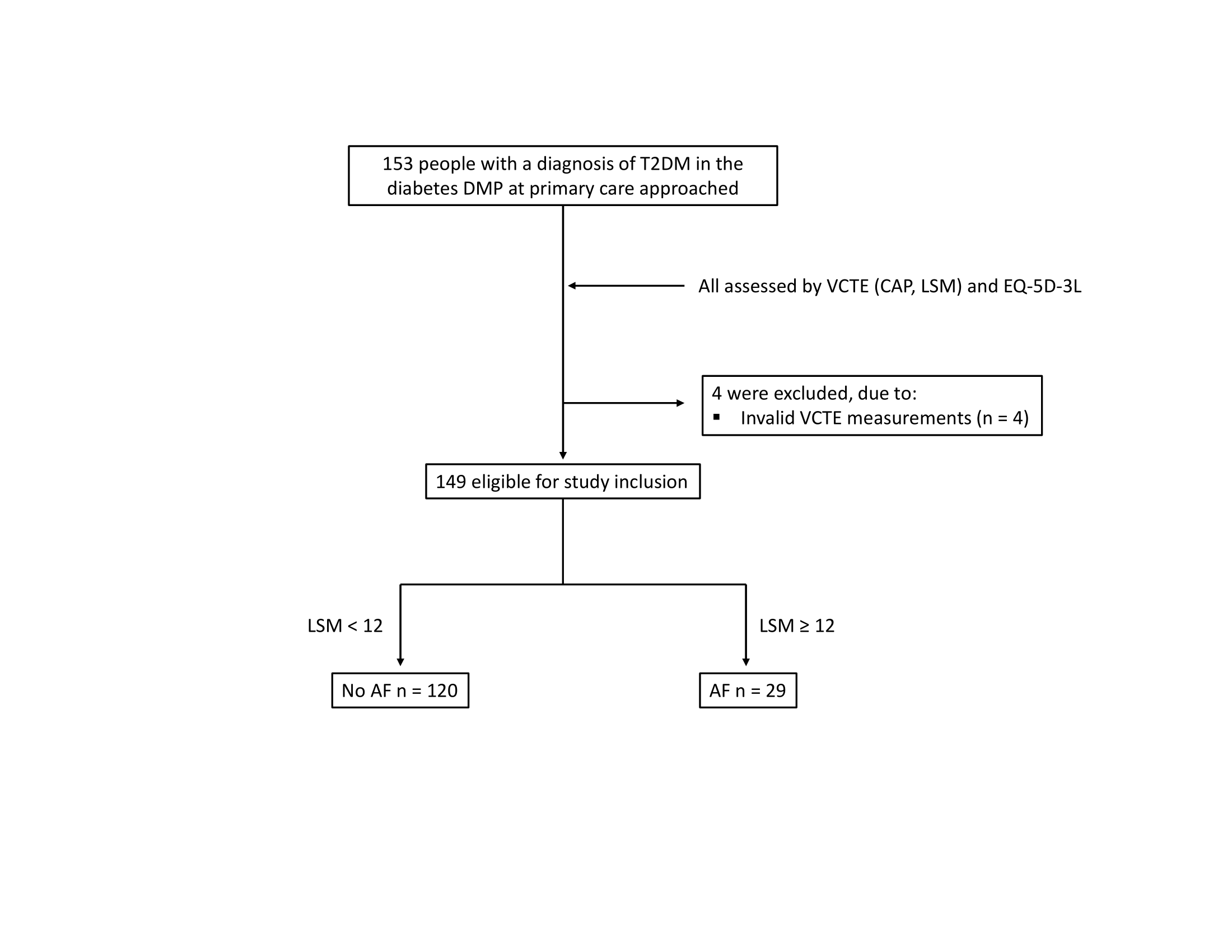

Supplement: Supplementary file 1 — Supplementary Figure 1. [file 41598_2024_72105_MOESM1_ESM.tiff]
