# Supplementary material for: Impairment of health-related quality of life among people with type 2 diabetes and advanced liver fibrosis
Source: Sci Rep. 2024 Sep 17;14:21650. doi: 10.1038/s41598-024-72105-8 (PMC11408596; doi:10.1038/s41598-024-72105-8)
Supplement: Supplementary file 2 — Supplementary Tables. [file 41598_2024_72105_MOESM2_ESM.docx]

**Supplement**

**Supplementary Table 1 Demographics, clinical characteristics, and comparison between males and females**

| **Variable** | **Males**  **(n = 94, 63.1%)** | **Females**  **(n = 55, 36.9%)** |  | **Effect sizes** |
| --- | --- | --- | --- | --- |
|  | **n (% or IQR)** | **n (% or IQR)** | ***p-value*** | **Cohen’s d or w** |
| **General characteristics** |  |  |  |  |
| Age in years | 67.0 (59.0; 71.0) | 65.0 (57.0; 70.0) | 0.098 | 0.335 S |
| Time since diagnosis | 12.0 (5.0; 19.0) | 10.0 (6.0; 16.0) | 0.191 | 0.258 S |
| History of cancer | 5 (5.3) | 8 (14.5) | 0.054 | 0.158 S |
| Harmful alcohol consumption  (AUDIT ≥ 8) n = 148 | 11 (11.8) | 1 (1.8) | **0.031** | -0.177 S |
| **VCTE** |  |  |  |  |
| CAP (dB/m) | 337.0 (276.3; 371.0) | 328.0 (291.0; 371.0) | 0.975 | -0.068 N |
| SLD (CAP ≥ 275 dB/m) | 71 (75.5) | 45 (81.8) | 0.373 | 0.073 N |
| MASLD n = 147 | 60 (65.2) | 44 (80.0) | 0.057 | 0.157 S |
| LSM (kPa) | 7.0 (5.8; 9.9) | 7.6 (5.6; 11.9) |  | -0.165 N |
| AF | 16 (17.0) | 13 (26.6) | 0.325 | 0.081 N |
| **Metabolic comorbidities** |  |  |  |  |
| BMI (kg/m²) | 31.5 (27.5; 35.3) | 31.8 (27.9; 37.0) | 0.382 | -0.270 S |
| Obesity (> 30 kg/m²) | 57 (60.6) | 32 (58.2) | 0.768 | -0.024 N |
| Waist circumference (cm) | 111.0 (102.0; 124.0) | 108.0 (97.0; 120.0) | 0.172 | 0.177 N |
| Hyperlipidemia | 37 (39.4) | 19 (34.5) | 0.558 | -0.048 N |
| Arterial hypertension | 84 (89.4) | 50 (90.9) | 0.762 | 0.025 N |
| MetS | 57 (60.6) | 32 (58.2) | 0.768 | -0.024 N |
| **Laboratory values** |  |  |  |  |
| GGT (U/l) n = 148 | 38.0 (29.5; 58.5) | 35.5 (24.0; 56.3) | 0.376 | -0.102 N |
| TG Triglycerides (mg/dl) n=147 | 157.0 (106.5; 241.0) | 186.0 (116.0; 267.8) | 0.144 | 0.065 N |
| TC (mg/dl) n= 148 | 179.0 (145.0; 209.3) | 216.0 (177.3; 247.0) | **0.001** | -0.777 M |
| Platelets (/nl) n = 148 | 214.0 (177.8; 257.0) | 232.5 (211.0; 297.5) | **0.010** | -0.401 S |
| HbA1c (%) n = 148 | 6.9 (6.5; 7.5) | 7.1 (6.4; 8.3) | 0.215 | -0.266 S |
| **T2DM-related comorbidities** |  |  |  |  |
| CVD | 36 (38.3) | 19 (34.5) | 0.647 | -0.038 N |
| CKD | 34 (36.2) | 15 (27.3) | 0.265 | -0.091 N |
| Polyneuropathy | 36 (38.3) | 11 (20) | **0.020** | -0.190 S |
| Retinopathy | 11 (11.7) | 3 (5.5) | 0.207 | -0.103 S |
| DFS | 15 (19.5) | 4 (7.3) | 0.125 | -0.126 S |
| **T2DM-related medication** |  |  |  |  |
| Metformin | 63 (52.7) | 29 (52.7) | 0.083 | -0.142 S |
| Insulin | 46 (48.9) | 29 (52.7) | 0.655 | 0.037 N |
| SGLT2 inhibitor | 16 (17.0) | 6 (10.9) | 0.310 | -0.083 N |
| No treatment | 6 (6.4) | 10 (18.2) | **0.025** | 0.184 S |

Abbreviations: AF, advanced fibrosis; AUDIT, Alcohol Use Disorders Identification Test; VCTE, vibration-controlled transient elastography; CAP, controlled attenuation parameter; MASLD, metabolic dysfunction-associated steatotic liver disease; LSM, liver stiffness measurement; BMI, body mass index; MetS, metabolic syndrome; GGT, gamma-glutamyl transferase; SLD, steatotic liver disease; TG, triglycerides; TC, total cholesterol; CVD, cardiovascular disease; CKD, chronic kidney disease; DFS, diabetic foot syndrome. Effect sizes: N, null; S, small; M, medium. Data are expressed as numbers, median, percentage (%), or interquartile ranges (IQR 25th; 75th). Boldface indicates statistical significance. A p-value < 0.05 was considered statistically significant.

**Supplementary Table 2 Comparison of HRQL between males and females**

| **Variable** | **Males (n = 94)** | **Females (n = 56)** | ***p-value*** | **Effect sizes**  **Cohen’s d** |
| --- | --- | --- | --- | --- |
| **EQ-5D-3L** |  |  |  |  |
| Mobility n = 148 | 1.27 ± 0.44 | 1.31 ± 0.51 | 0.646 | -0.084 N |
| Self-care n = 149 | 1.04 ± 0.20 | 1.05 ± 0.23 | 0.739 | -0.046 N |
| Usual activities n = 149 | 1.18 ± 0.39 | 1.24 ± 0.43 | 0.416 | -0.146 N |
| Pain/discomfort n = 147 | 1.68 ± 0.57 | 1.72 ± 0.68 | 0.839 | -0.064 N |
| Anxiety/depression n = 149 | 1.20 ± 0.49 | 1.34 ± 0.55 | **0.043** | -0.269 S |
| VAS n = 143 | 70.6 ± 18.5 | 74.2 ± 18.0 | 0.265 | -0.197 N |
| TTO index n = 146 | 0.85 ± 0.19 | 0.83 ± 0.24 | 0.783 | 0.092 N |

Data are expressed as means with standard deviation. Boldface indicates statistical significance. A p-value < 0.05 was considered statistically significant. Effect sizes: N, null; S, small.

**Supplementary Table 3** **Comparison of HRQL between no SLD and SLD**

| **Variable** | **No SLD (n = 33)** | **SLD (n = 116)** | ***p-value*** | **Effect sizes**  **Cohen’s d** |
| --- | --- | --- | --- | --- |
| **EQ-5D-3L** |  |  |  |  |
| Mobility n = 148 | 1.12 ± 0.33 | 1.33 ± 0.49 | **0.023** | -0.503 M |
| Self-care n = 149 | 1.03 ± 0.17 | 1.05 ± 0.22 | 0.609 | -0.102 N |
| Usual activities n = 149 | 1.06 ± 0.24 | 1.24 ± 0.43 | **0.023** | -0.517 M |
| Pain/discomfort n = 147 | 1.50 ± 0.57 | 1.75 ± 0.62 | **0.044** | -0.420 S |
| Anxiety/depression n = 149 | 1.15 ± 0.36 | 1.28 ± 0.56 | 0.275 | -0.276 S |
| VAS n = 143 | 74.6 ± 18.9 | 71.1 ± 18.2 | 0.291 | 0.189 N |
| TTO index n = 146 | 0.92 ± 0.13 | 0.83 ± 0.22 | **0.037** | 0.498 S |

Data are expressed as means with standard deviation. Boldface indicates statistical significance. A p-value < 0.05 was considered statistically significant. Effect sizes: N, null; S, small; M, medium.

**Supplementary Table 4** **Comparison of HRQL between no obesity and obesity**

| **Variable** | **No obesity (n = 60)** | **Obesity (n = 89)** | ***p-value*** | **Effect sizes**  **Cohen’s d** |
| --- | --- | --- | --- | --- |
| **EQ-5D-3L** |  |  |  |  |
| Mobility n = 148 | 1.17 ± 0.38 | 1.36 ± 0.51 | **0.013** | -0.422 S |
| Self-care n = 149 | 1.05 ± 0.22 | 1.04 ± 0.21 | 0.887 | 0.046 N |
| Usual activities n = 149 | 1.10 ± 0.30 | 1.27 ± 0.45 | **0.012** | -0.444 S |
| Pain/discomfort n = 147 | 1.44 ± 0.53 | 1.86 ± 0.61 | **< 0.001** | -0.735 M |
| Anxiety/depression n = 149 | 1.15 ± 0.36 | 1.33 ± 0.59 | 0.086 | -0.368 S |
| VAS n = 143 | 78.5 ± 13.5 | 67.6 ± 19.7 | **0.001** | 0.645 M |
| TTO index n = 146 | 0.92 ± 0.12 | 0.79 ± 0.24 | **< 0.001** | 0.685 M |

Data are expressed as means with standard deviation. Boldface indicates statistical significance. A p-value < 0.05 was considered statistically significant. Effect sizes: N, null; S, small; M, medium.
